# MRI perfusion in evaluating ovarian masses: diagnostic performance of wash out rate

**DOI:** 10.3389/fonc.2026.1609090

**Published:** 2026-05-28

**Authors:** Heba Sabet, Moawia Gameraddin, Fahad H. Alhazmi, Walaa Alsharif, Nagham N. Omar, Ahmed Sobh, Sultan A. Alshoabi, Awadia Gareeballah, Maisa Elzaki, Hadeel A. Ghunaim, Awatif M. Omer, Amel F. H. Alzain, Reem M. Elkady

**Affiliations:** 1 Department of Radiology, Faculty of Medicine, Assiut University, Asyut, Egypt; 2 Department of Diagnostic Radiology, College of Applied Medical Sciences, Taibah University, Al-Madinah Al-Munawarah, Saudi Arabia; 3 Department of Obstetric & Gynecology Faculty of Medicine Assiut University, Asyut, Egypt; 4 Department of Internal Medicine, College of Medicine Taibah University, Al-Madinah Al-Munawarah, Saudi Arabia

**Keywords:** MRI, ovarian tumors, perfusion, semiquantitative, wash out rate

## Abstract

**Background and objectives:**

Preoperative Imaging evaluation of an ovarian lesion enables the surgeon to anticipate malignant lesions before the operation and plan an adequate operative procedure. In this study we assessed the role of semiquantitative MR perfusion parameters including wash-out rate in differentiating benign from malignant ovarian masses.

**Patients and methods:**

A prospective study of 82 indeterminate adnexal lesions on ultrasound basis. According to the inclusion criteria 29 patients with 32 adnexal lesions underwent conventional MRI sequences followed by MR perfusion technique based on a Dynamic Contrast Enhanced (DCE) 3D GE T1 high resolution isotropic volumetric examination (THRIVE). The conventional sequences were used to evaluate the lesion’s morphological criteria by two independent readers. Then tumor semi-quantitative enhancement parameters were calculated by computer software including Wash in Rate (WIR), wash out Rate (WOR), Maximum absolute enhancement (SI max), Maximum Relative Enhancement (SI rel), and Time to Peak (TTP). Morphological findings and calculated parameters were correlated with the histopathological results.

**Results:**

A significant correlation was found between pathological diagnosis and T2 signal intensity, ascites and peritoneal deposits where predicative features of malignancy were isointense signal on T2 (p value0.028), presence of ascites (p value 0.008), and peritoneal deposits (p value 0.022). For the correlation between the Semi quantitative perfusion MRI parameter and the pathological diagnosis the WOR in the study group ranged from 0.06 to 68.84 with mean value of 12.6 ± 17.46. Both SI max and WOR were able to significantly differentiate benign and malignant lesions (P = 0.017*&0.028* respectively). Applying cutoff value of WOR >6.03 provided accuracy of 86.2% with 82.4% sensitivity, 90% specificity, 93.3% Positive Predictive value (PPV) and 75% Negative Predictive value (NPV). While applying a cutoff value of SI max >1391.07 provided accuracy of 73.2% with 76.5 sensitivity, 70% specificity,81.2% PPV and 63.6% NPV.

**Conclusion:**

The addition of DCE MR Sequence to the conventional MRI has improved its diagnostic accuracy. we can depend upon WIR in depicting malignant masses and correct our diagnosis by applying the cutoff of WOR to exclude the false positive cases and avoid unnecessary radical surgery especially in women aiming at preserving fertility.

## Introduction

Ovarian masses are frequently found in clinical practice either in symptomatic patients or incidentally detected. Characterization of an ovarian lesion represents a diagnostic dilemma considering the wide differential diagnosis and the importance of accurate diagnosis in proper management (1). Preoperative Imaging evaluation of an ovarian lesion enables the surgeon to anticipate malignant lesions before the operation and plan an adequate operative procedure (2).This is important for young women who wish to preserve fertility where a conservative surgery should be offered and for elderly women where a confident preoperative characterization may save them from the hazards of radical surgery (3) Ultrasonography (US) is the first-step imaging technique for assessing the origin and characteristics of pelvic masses (4). Despite using algorithms taking into account clinical manifestations, CA125 serum level, and ultrasonographic findings preoperative characterization remains difficult for complex lesions with up to 25% of adnexal masses remain indeterminate on US and require further imaging evaluation (5, 6). The conventional Magnetic resonance imaging (MRI) examination based on morphological and signal intensity appearances is proven to generate higher accuracy with US. Yet nonspecific imaging features and dependence on experience of the reader restrict the liability of conventional MRI (7). Therefore, functional MR techniques have been incorporated in preoperative characterization of female pelvic masses (8). The technique of utmost importance for characterization of complex adnexal masses is semi quantitative multiphase-dynamic contrast-enhanced MRI where the contrast uptake in adnexal masses is determined by the tumor biological process and the presence of neo angiogenesis (9, 10). MR contrast agents can pass more quickly through tumor vessels to produce differential enhancement. This results in a fast “wash-in” of contrast coupled with the rapid “wash-out” that allows a functional analysis of the tumor microcirculation (11, 12). Tumor contrast wash out has been discussed in different body organs. However, limited publications discussed the role of wash-out parameters in differentiating benign from malignant ovarian masses. The aim of this study is to assess the role of semiquantitative MR perfusion parameters including wash-out rate (WOR) in differentiating benign from malignant ovarian masses.

## Materials and methods

**Figure 1:**
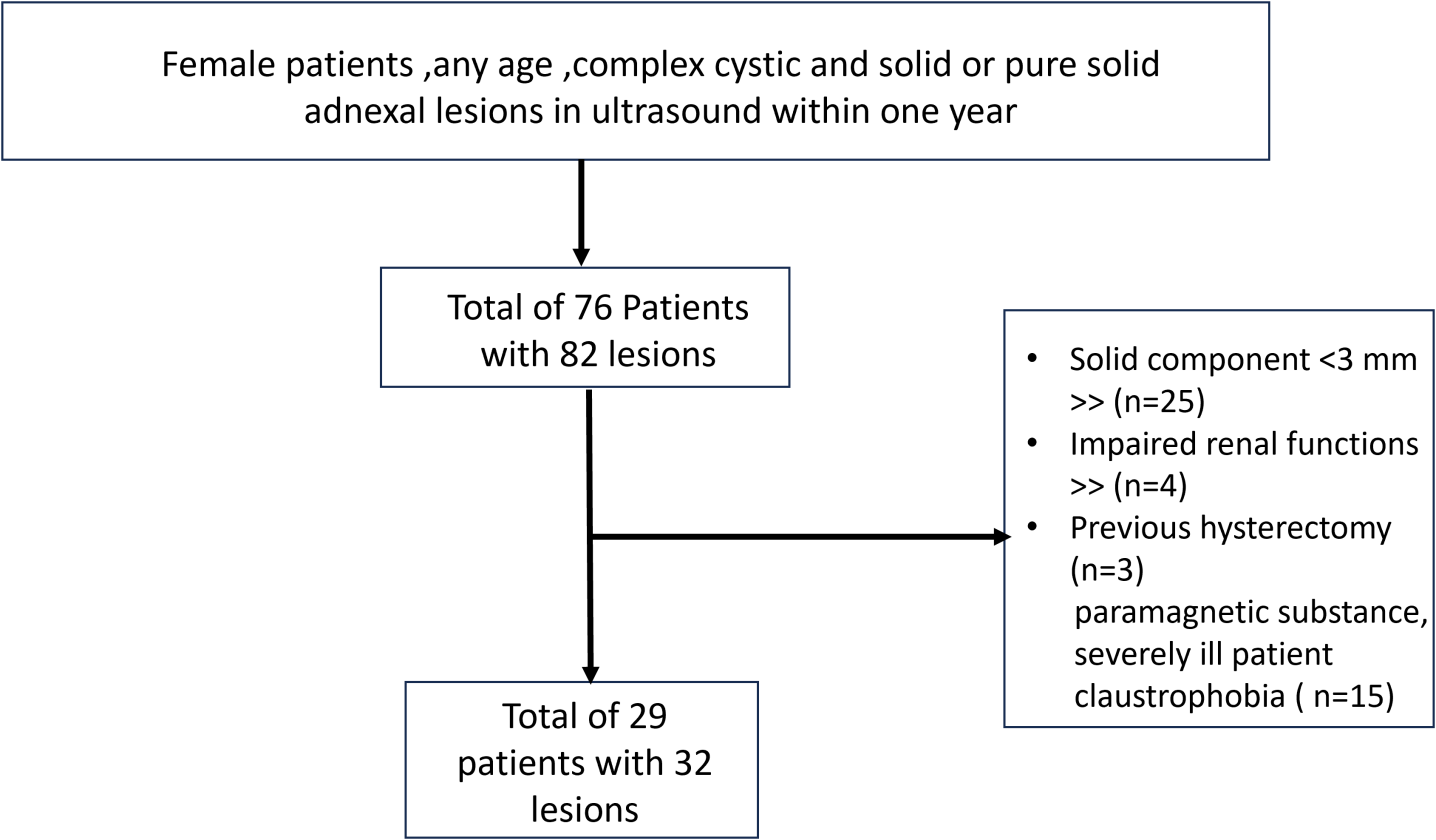
Flowchart for inclusion and exclusion criteria.

This is a prospective study approved by the institutional review board. An informed consent was obtained with respect to patients’ confidentiality. The study was conducted in the radiology department of our university hospital. 76 patients with 82 lesions were referred to our department from the university Women’s Health gynecological clinic within a year period. All the referred lesions were defined as indeterminate pelvic lesions on US basis. Our inclusion criteria in this study were female patients of different age groups with complex cystic and solid or pure solid adnexal lesions. The solid component (vegetations, thick septa or soft tissue) should be of sufficient size ≥3mm to draw a region of interest (ROI).The lesions with simple cystic adnexal lesions or contain fat component were excluded from our study. The Lesions that had a solid component <3 mm were excluded as it was not possible to obtain meaningful post -processing data. Patients with impaired renal functions and those who underwent previous hysterectomy were excluded. Patients with any general contraindication to MRI as presence of paramagnetic substance, severely ill patients or those with claustrophobia were excluded. A total of 29 patients with 32 Adnexal lesions were finally included in our study. A flow chart of inclusion and exclusion criteria is shown in **Figure 1**.

### MR imaging technique

MR imaging was performed on 1.5 Tesla MR scanner (Achievia, Philips Healthcare, Netherlands) All the patients were imaged in the supine position using pelvic phased-array coil. The examination was conducted on the female pelvis including conventional MRI sequences; axial, sagittal and coronal T2WI, axial T1WI and pre contrast axial T 1 Fat suppression. Followed by perfusion weighted imaging (PWI). All sequences were performed with saturation bands placed anteriorly and posteriorly to eliminate artifacts induced by the high signal from subcutaneous fat.MR perfusion technique was based on a Dynamic Contrast Enhanced (DCE) 3D GE T1 high resolution isotropic volumetric examination (THRIVE). The imaged stack should include part of the soft tissue inside the adnexal mass (i.e., solid portion, papillary projections, or thickened irregular septa) and the adjacent external myometrium in the optimal plane. Axial plane was performed in all cases except one case where we had to obtain the sequence in oblique Sagittal plane in order to include the solid tissue and myometrium in the imaged stack. Gadolinium chelate (Dimeglumine gadopentetate, Magnevist; Germany) was given at a dose of 0.2 ml per kilogram of body weight by using a power injector (Medrad, spectris solaris) at a rate of 2 ml/sec, followed by 20 ml of normal saline. Imaging started 14 seconds before the bolus injection for a total of 420 seconds (7 minutes) and the images were obtained sequentially every 14 seconds. Forty sequential slices were acquired with slice thickness 2 mm. MRI scan protocol is shown in (**Table 1**).

**Table 1:** MRI scan protocol.

Sequence	TR	TE	Slice thickness	Intersection gap	Matrix size
Saggital T2	3000	120	4 mm	0 mm	180x158
Axial T2	4154	120	6 mm	0 mm	180x170
Coronal T2	3000	120	4 mm	0 m	180x158
AXIAL T1	534	10	6 mm	0 mm	184x159
Axial T1 SPIR	455	8.0	5 mm	5 mm	260x223
Axial PWI (3D FFE T1)	4.0	1.93	2 mm	0 mm	124x118

### Image interpretation

The images were transferred to Philips MR workspace. The conventional sequences were applied to evaluate the lesion’s morphological criteria, including the lesion laterality, size, T2 signal intensity of the solid part inside the mass relative to the outer myometrium and finally presence of ascites and/or peritoneal disease. Imaging findings were reviewed by 2 radiologists with more than 15 years’ experience. Both readers interpret MRI images independently and blindly to clinical information. For perfusion images three types of analysis were performed: first is Visual (Qualitative) analysis of sequential enhancement of the solid parts of the lesion relative to myometrial enhancement on phases of dynamic contrast enhanced sequence. The second analysis is the time intensity curve analysis. For adnexal mass characterization, the region of interest (ROI) placement was conducted manually by both readers. For precise calculation the ROI dimension should be equal or exceed 3 mm^2^. for each lesion, two (ROI) were placed on the external myometrium and on the most enhancing part of the solid tissue of the adnexal mass using maximum enhancement colored map generated in the workstation.

The enhancement of the solid tissue was classified according to the previously published time–signal intensity curve classification (11):

- Type 1: A gradual increase in the signal intensity of the solid tissue, without a well-defined “shoulder”.
- Type 2: A moderate initial increase in the signal intensity of solid tissue relative to that of myometrium, followed by a plateau.
- Type 3: An initial increase in the signal intensity of solid tissue that was steeper than that of myometrium.

Finally, the tumor semi-quantitative enhancement parameters were calculated by computer software. In this study the following parameters were calculated: Wash in Rate [l/s] (WIR), Wash out Rate [l/s] (WOR), Maximum absolute enhancement (SI max), Maximum Relative Enhancement [%] (SI rel), Time to Peak (TTP). Semiquantitative analysis parameters are demonstrated in **Supplementary File S1**. Morphological findings and calculated parameters were correlated with the histopathological results attained by the surgical specimens.

### Statistical methods

Statistical analysis was performed using the SPSS^®^ software for Windows v. 20 (SPSS Inc., Chicago, IL. An inter-rater agreement for each test was calculated using Cohen’s kappa coefficient (j) which is calculated as follows: j = (p0 pe)/(1 pe), where p0 is the observed proportion of agreement and pe is the expected proportion of agreement. Data were statistically described in terms of range, mean ± SD if they are parametric and median ± range if they were nonparametric. For comparing qualitative data, chi square was performed. For comparing quantitative data, T-test was performed in parametric data and Mann-Whitney test in non-parametric data. Accuracy of the studied diagnostic test in predicting malignancy was represented using the terms sensitivity, specificity. Overall accuracy, negative predictive values (NPV) and positive predictive values (PPV). A probability value (p=0.05) was considered statistically significant. Receiver operating characteristic curve (ROC) were used to determine the cutoff of semi quantitative parameters.

### Ethical considerations

All procedures were performed in compliance with relevant laws and institutional guidelines and were approved by our institutional review board (04-2023-200596). Patients’ privacy and confidentiality were respected and Informed consent was obtained.

## Results

Overall, 29 female patients with 32 adnexal lesions were included in this study (age range, 15 to 76 years; mean age,44.41 ± 19.2 years). Twenty-one (72.4%) out of 29 patients were in premenopausal status. The main presenting symptom was abdominal pain in 26 (89.6%) out of 29 cases. No statistically significant correlation was found between age, menopausal status, and presenting symptom with the pathological diagnosis.

All the cases underwent surgical management; 17 cases underwent hysterectomy with bilateral salpingeo-oopherectomy, 8 cases underwent simple oophorectomy, and 4 cases underwent ovarian cystectomy. Regarding histopathological results, 15 (47%) were malignant ovarian masses, 2 (6%) were borderline ovarian masses and 15 (47%) were benign ovarian masses. The result of the pathological diagnosis of the 32 adnexal lesions is listed in **Table 2**. As regards the interrater reliability in this study, there was almost perfect agreement in the assessment of MRI images between the two readers (0.94).

**Table 2:** Histopathological results of adnexal lesions in 32 women.

Pathology	Benign (n=15)	Malignant/borderline (n=17)	Total (n=32)
**Epithelial**	Serous = 4 (12.5%) Mucinous = 2 (6.2%)	Serous = 6 (18.7%) Endometroid = 1 (3.1%) Borderline serous = 2 (6.2%)	15 (47%)
**Sex cord stromal**	Fibroma/thecoma = 6 (18.7%) Benign sclerosing stromal tumor of the ovary = 1 (3.1%)	Adult granulosa cell tumor = 5 (15.6%)	12 (37.5%)
**Germ cell tumor**		Immature teratoma = 1 (3.1%)	1 (3.1%)
**Ovarian metastasis**		Metastasis = 2 (6.2%)	2 (6.2%)
**Inflammatory**	Tubo-ovarian abscess = 2 (6.2%)		2 (6.2%)

The 32 adnexal masses were analyzed using conventional MRI sequences according to their diameters, morphological features including presence of solid tissue (cystic, solid, mixed cystic and solid lesions), T2 signal of solid tissues, presence of ancillary features as ascites, peritoneal deposits and lymphadenopathies. The mean size of ovarian masses was 13.86 ± 7.49 cm (range: 3–29 cm). A significant correlation was found between pathological diagnosis and T2 signal intensity, ascites and peritoneal deposits where predicative features of malignancy are isointense signal on T2 (p value0.028), presence of ascites (p value 0.008), and peritoneal deposits (p value 0.022) (**Table 3**). No significant correlation was found between the largest dimension and pathological diagnosis.

**Table 3:** The relation between conventional criteria of all 32 adnexal lesions and the pathological findings.

Conventional Criteria	Malignant		Benign		P value
	No.	%	No.	%	
Soft tissue component					
Yes	17/17	100.0	15/15	100.0	–
T2 signal					
Iso	16/17	94.1	8/15	53.3	0.028*
Hypo	1/17	5.9	6/15	40.0	
Hyper	0/17	0.0	1/15	6.7	
Ascites					
Yes	16/17	94.1	8/15	53.3	0.008**
No	1/17	5.9	7/15	46.7	
Peritoneal deposits					
Yes	5/17	29.4	0/15	0.0	0.022*
No	12/17	70.6	15/15	100.0	

• P < 0.05 significant.

• ** P< 0.01 highly significant.

For time intensity curve analysis, Type 1 curve was detected in 8 (25%) out of 32 examined lesions, all of them proved to be benign lesions, while type 2 curve was depicted in 7 (21.8%) lesions, 4 of them were borderline or malignant and type 3 was detected in 17 (53.2%) lesions 13 of them were malignant.

**Figure 2:**
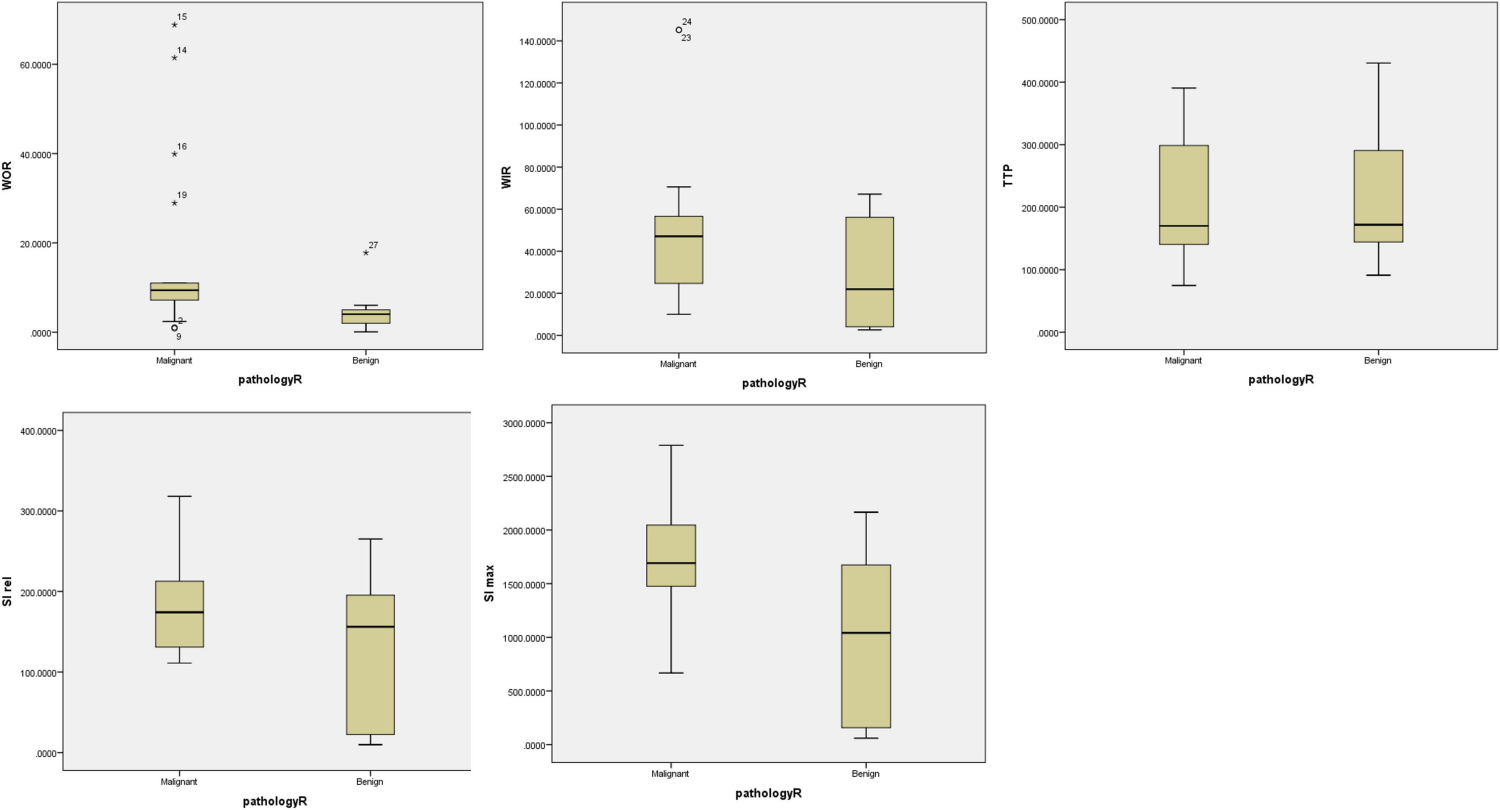
Box plot diagrams of perfusion weighted MRI parameter.

For the semi quantitative analysis, the mean values of perfusion parameters in benign versus malignant lesions are illustrated in **Figure 2**. The WOR in the study group ranged from 0.06 to 68.84 with mean value of 12.6 ± 17.46. The range and mean values of different perfusion parameters of 32 ovarian lesions are shown in **Table 4**. For the correlation between the Semi quantitative perfusion MRI parameter and the pathological diagnosis both SI max and WOR were able to significantly differentiate between benign and malignant lesions (P = 0.017*&0.028* respectively) as shown in **Table 5**.

**Table 4:** The mean values of different perfusion parameters of 32 ovarian lesions.

Parameter	Range	Mean ± SD
SI max	59.94-2789.25	1465.79 ± 698.98
SI rel	9.84-318.09	166.66 ± 73.67
TTP	74.8-430.54	222.11 ± 108.45
WIR	2.6-145.21	43.7 ± 35.71
WOR	0.06-68.84	12.6 ± 17.46

**Table 5:** The relation between the Semi quantitative perfusion MRI parameter and the pathological diagnosis in 32 ovarian masses -

Parameters	Malignant	Benign	P value
Simax	1705.184 (+/544.3673)	1058.826 (± 769.6442)	0.017*
Sirel	182.57 (+/-58.03)	139.60 (+/-91.65)	0.147
TTP	221.10 (+/-108.09)	221.10 (+/-108.09)	0.951
WIR	52.36 (+/-39.11)	28.97 (+/-24.11)	0.101
WOR	17.19 (+/-20.52)	4.79 (+/-4.96)	0.028*

• * P value is significant.

**Figure 3:**
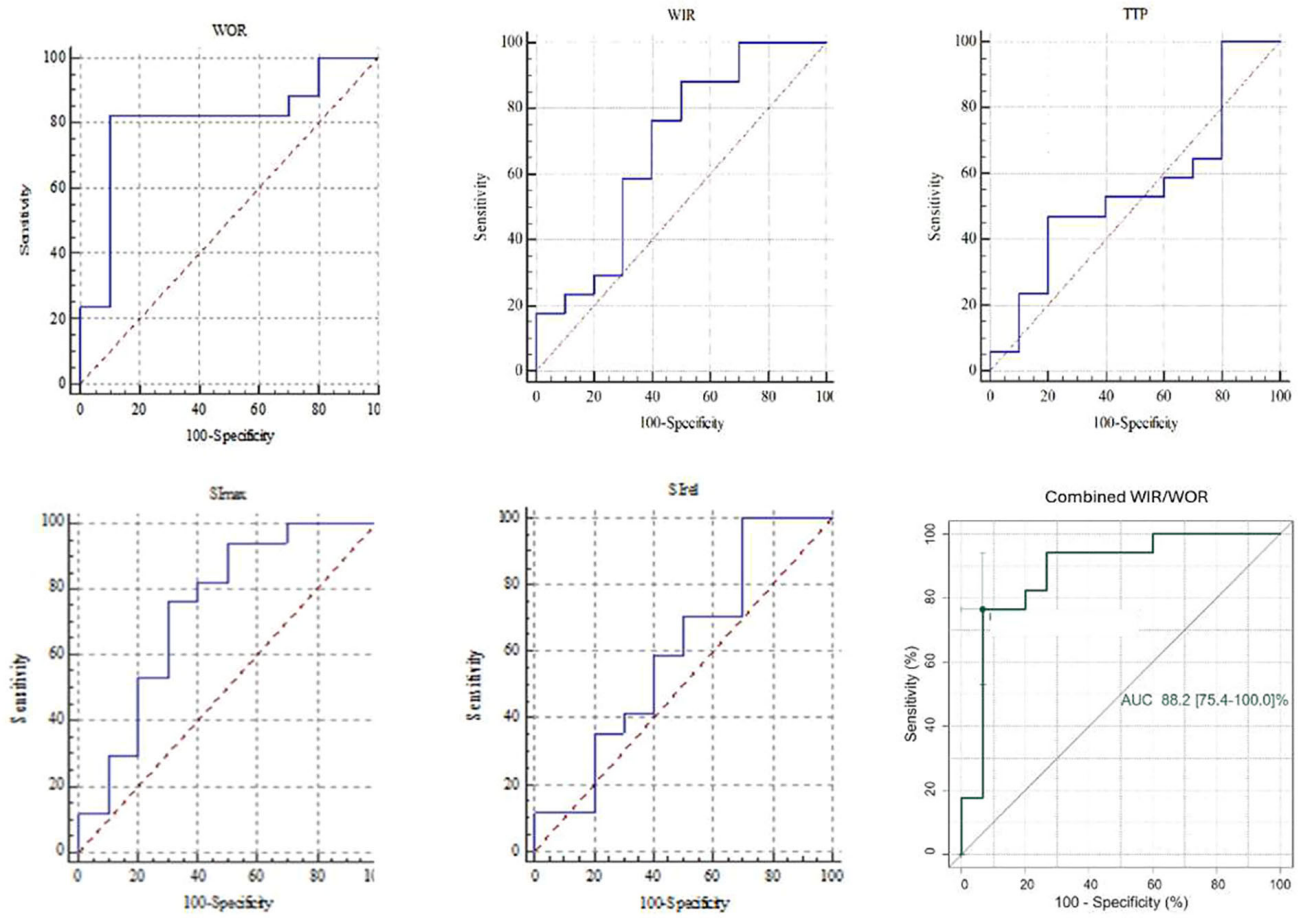
ROC curve analysis of PW-MRI parameters (WOR, WIR, TTP, SI max, SI rel, and combined WOR/WIR).

Further analysis was carried out to establish optimal threshold criteria for the prediction of malignancy. The ROC curve was constructed to evaluate the AUC for each individual perfusion parameter. The smallest overlap and the largest AUC were observed using WOR and SI max parameters (**Figure 3**); that provided the most reliable enhancement data for distinguishing between benign and borderline/invasive malignant lesions. Applying cutoff value of WOR >6.03 provided accuracy of 86.2% with 82.4% sensitivity, 90% specificity, 93.3% PPV and 75% NPV. while applying a cutoff value of SI max >1391.07 provided accuracy of 73.2% with 76.5 sensitivity, 70% specificity,81.2% PPV and 63.6% NPV. Combined ROC curve analysis was done for WIR and WOR parameters and provided accuracy of 88.2% with 93.3% sensitivity, and 76.5% specificity. The cutoff value of the perfusion MRI parameters for the benign versus malignant lesions with their accuracy, sensitivity, specificity positive and negative predictive values are shown in **Table 6**.

**Table 6:** The cutoff value of the perfusion MRI parameters for malignant versus benign lesions

Parameters	AUC	Cutoff *	Sensitivity	Specificity	PPV	NPV	Accuracy
Simax	0.74	>1391.07	76.5	70.0	81.2	63.6	73.2
Sirel	0.60	> 22.38	100.0	30.0	70.8	100.0	65.0
TTP	0.55	≤ 143.28	47.1	80.0	80.0	47.1	63.5
WIR	0.68	> 20.89	88.2	50.0	75.0	71.4	69.1
WOR	0.81	> 6.03	82.4	90.0	93.3	75.0	86.2
Combined WIR/WOR	0.88	–––	93.3	76.5	92.9	77.8	84.4

*For malignant lesions.

## Discussion

Ovarian tumors are the most prevalent reason for gynecologic surgery. The treatment plan relies on whether the tumor is benign, borderline, or invasive, therefore preoperative characterization is critical (11). This is especially true for young women, who should receive conservative surgery for fertility preservation (13). The aim of the current study is assessing the added role of perfusion MRI to conventional MRI in characterization of adnexal masses to optimize the surgical management of the patients thus improving patient’s outcome.

In this study rapid DCE acquisition (every 14 seconds) was performed for 30 consecutive phases with the whole time of the sequence of 7 minutes as recommended by Thomassin-Naggara et al. to increase the accuracy of the early enhancement parameters and allows derivation of accurate time intensity curves (14). We performed DCE for 7 minutes as Gundogdu et al. performed DCE on normal ovary and demonstrated that the percentage of washout in ovary was the highest at 5th minutes (15). This is contradictory to Bernardin et al., which performed a slow DCE sequence. They performed a 5-point dynamic run (0, 30, 60, 90,120 seconds). However, the reduction in temporal resolution inevitably reduces the accuracy of the early enhancement parameters (3).

In the current study we used the external myometrium as reference tissue as done by Thomassin-Naggara et al. The latter study found that there is no difference between the proliferative and the secretory phases, for all analyzed DCE parameters in the outer myometrium which makes it a suitable reference point. However, the outer myometrium of postmenopausal women displayed lower perfusion parameters than women of reproductive age and therefore this must be considered when enhancement curves are examined (16) Other authors used the pelvic striated muscles (psoas muscle) as reference tissue. However, striated muscles show variations in their perfusion in correlation with muscular atrophy (17).

The regions of interest in our cases were manually designated for the calculation of the semiquantitative parameters. While this method could enhance accuracy, it may compromise the reproducibility of the measurements. To address this issue, we engaged two radiologists in the image interpretation process and assessed the interrater reliability, which indicated almost perfect agreement. The recent adoption of radiomics analysis offers improved reproducibility using software for semiautomated selection of regions of interest. In the semiautomated segmentation process, the radiologist manually outlined the area of interest in the slice exhibiting the largest cross-sectional area. Subsequently, the software automatically extended the contours to all other slices, which the radiologist reviewed and modified to confirm accuracy (18). Moreover, the use of Textural features in radiomics takes into account voxels and their surrounding neighbors, offering a unique representation of tumor heterogeneity (19).

In the current study, Curve type 1 was found to be specific for benign ovarian tumors with 100% Specificity, while type II curve showed significant overlap between benign and malignant tumors with no evident diagnostic performance. Meanwhile, type III was detected in 17 (53.2%) cases, 4 of them were benign with sensitivity (76%) and Specificity (60%). Among the four benign cases, two cases were benign sclerosing stromal ovarian tumor and fibrothecoma that showed ectatic blood vessels on histopathological analysis which explains the enhancement pattern giving rapid rise in signal intensity that is steeper than that of myometrium with well-defined shoulder. The other benign cases were tubo-ovarian abscess where type III curve could be explained by hyperemia and congestion of the regional vessels. Our results matched with Thomassin-Naggara et al. that reported that Curve type 3 is specific for invasive tumors with a sensitivity of 67% with overlap among the curve types, reducing their capability to differentiate between benign and malignant tumors (12). Hence the importance of combining time intensity curve analysis (TIC) with semiquantative perfusion parameters. The suggested pathophysiology of tumor growth assumes that tumors induce angiogenesis to grow. However, angiogenic vessels formed by the tumors have substantial gaps between the endothelial cells as well as between the basement membrane and the pericytes, which makes the vessels hyperpermeable to many macromolecules (20), These properties can be utilized by DCE-MRI. MR contrast agents that pass slowly through the normal vasculature can travel more quickly through tumor vessels, creating differential enhancement. This leads to a fast “wash-in” of the contrast coupled with a rapid “wash-out,” allowing a functional analysis of the tumor microcirculation (21).

**Figure 4:**
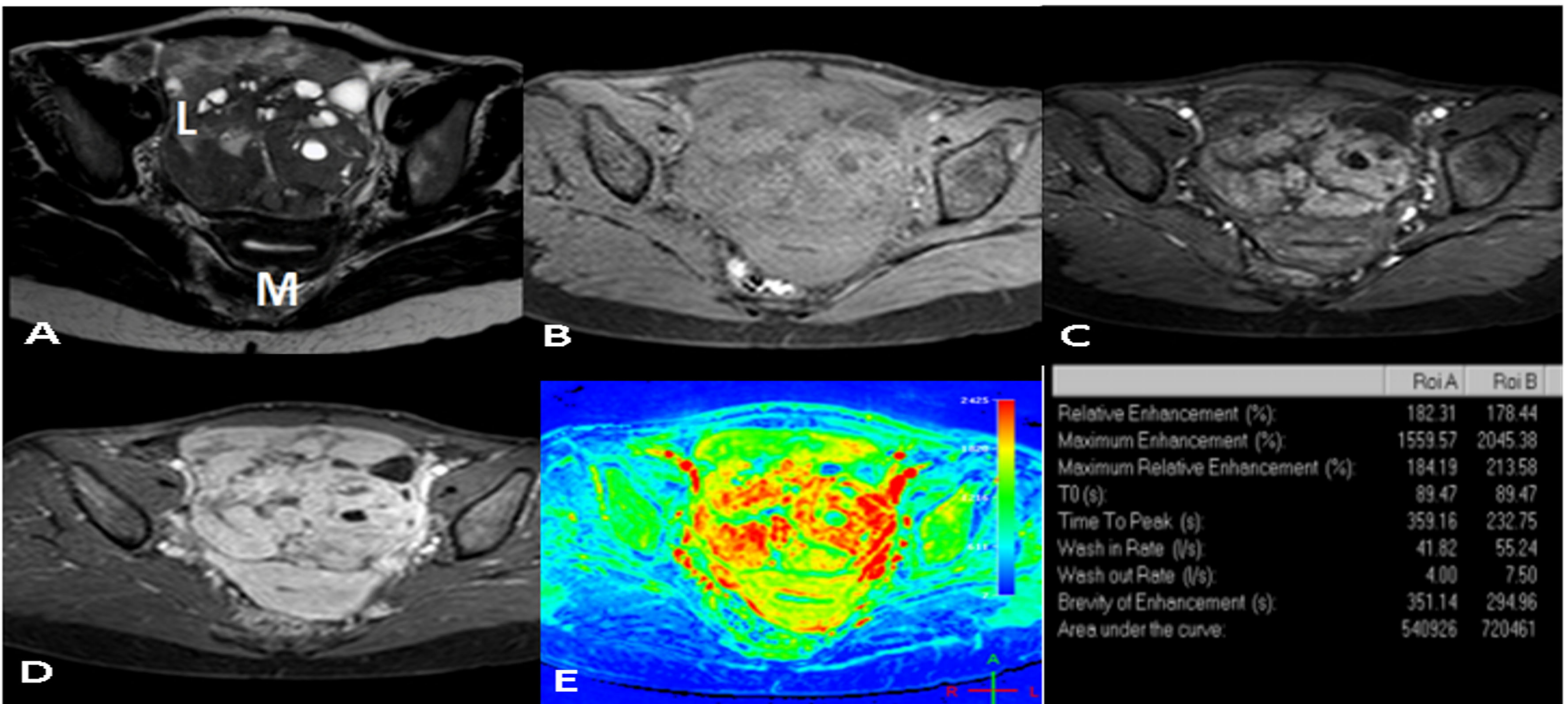
Thirty-three years old female patient with abdominal mass. Axial T2WI **(A)** shows heterogenous cystic and solid lesion with minimal ascites. Axial 3D T1fat suppression dynamic contrast MR **(B)** first non-contrast phase, **(C)** third dynamic phase, **(D)** fourth dynamic phase show early enhancement of the solid part within the ovarian lesion even before enhancement of the adjacent uterine myometrium. Colored map **(E)** shows red and yellow areas inside the solid parts which indicate areas of maximum enhancement. The wash in rate and wash out rate of the solid tissue (ROI B) is higher relative to that of the myometrium (ROI A) indicating malignant nature of the lesion. Pathological diagnosis was malignant sex cord tumor.

Many previous studies confirmed the value of WOR in differentiating malignant from benign lesions regardless of tumor origin (22–29). For instance, Liberman et al. reported a washout kinetic pattern in 70% of infiltrating breast carcinomas applying visual assessment of kinetic features on DCE-MRI (22). Yet few studies pointed out the efficacy of WOR in differentiating benign from malignant adnexal lesion. In 2003, Sohaib et al. investigated the increase in signal intensity of the solid components of the adnexal masses at 60 and 120 seconds of enhancement. They discovered that malignant lesions exhibit more enhancement than benign lesions during the early phase of enhancement rather than the late phase of enhancement (30). In the current study, the perfusion parameter that showed the most significant difference between benign and malignant lesions was WOR. We demonstrated that a lesion with a WOR > 6.03 is likely malignant (**Figure 4**) with accuracy 86% and PPV 93.3%. This was applied to all lesions in our study apart from tubo-ovarian abscess that showed higher value that could be explained by the effect of hyperemia. This is consistent with the findings reported by El ameen et al. The cutoff value for WOR in their study was > 6 for malignant lesions with PPV 100% (11). In another study done by Gity et al. they reported no significant difference between benign and malignant lesions using WOR with AUC = 0.49 (31). Their results can be explained by the distribution of pathology among their cases as they included a significant number of endometriomas which may show angiogenesis of the wall. All the previous demonstrate the importance of SI max depicted in early dynamic phases in discriminating between benign and malignant lesions. In the current study SI max showed relevant criterion for distinguishing invasive from non-invasive tumors. A cut off value >1391.07 yields 73.2% accuracy and PPV 81.2% in distinguishing benign and malignant tumors. Dilks et al. suggested a threshold value of SI max ≥ 250 for the prediction of malignancy producing a sensitivity of 100% and Specificity of 100% (17) In our study the SImax showed higher cut-off value compared to the later study, this could be explained by the different pathological distribution between both studies. Some of our benign cases (fibroma, thecoma, benign sclerosing stromal tumors with ectatic vessels at the level of microscopic picture and tubo-ovarian abscesses with associated hyperemia) showed high SI max values raising the cut off value.

**Figure 5:**
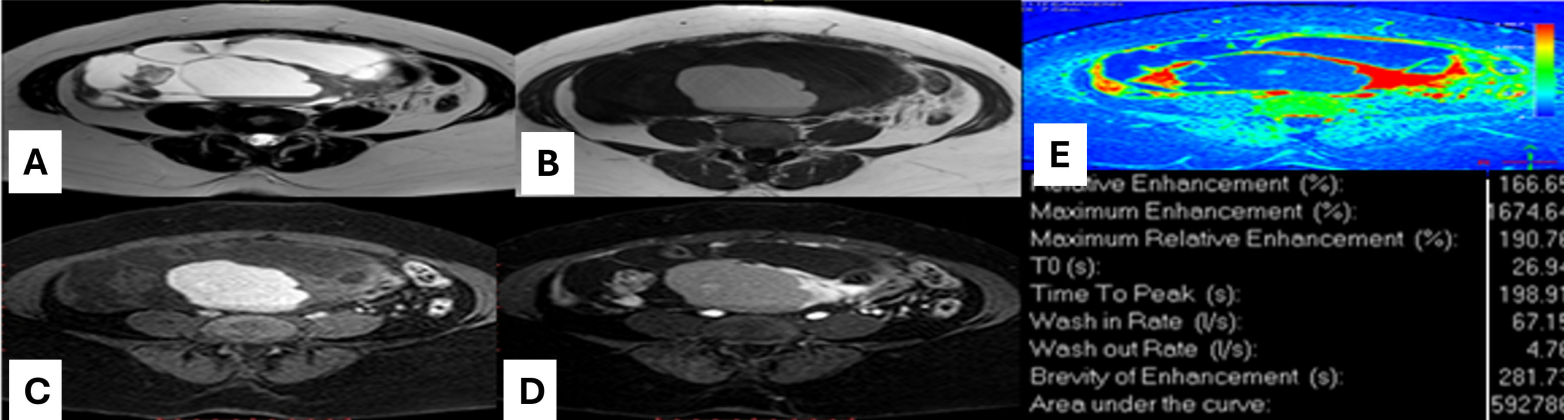
Thirty Years old female patient presented with abdominal swelling. Axial T2 and T1WI **(A, B)** show large pelviabdominal multilocular cystic lesion with thick septations and soft tissue components. It shows areas of hemorrhage on T1 weighted images. No ascites was depicted and no evidence of peritoneal deposits. Axial 3D T1 Fat suppression dynamic MR sequences **(C)** first non-contrast **(D)** fourth pass sequence and **(E)** colored map of maximum enhancement show early enhancement of the solid tissue relative to the myometrium. The solid parts show type 3 curve enhancement pattern (not included). MR perfusion parameters show high maximum enhancement and maximum relative enhancement of the solid tissue with shorter time to peak, and rapid wash in while the wash out rate of the solid was 4.76 (less than the suggested cut-off value of malignancy) suggesting benign nature. Pathological diagnosis was Benign sclerosing stromal tumor.

WIR and SI rel did not yield significant results in characterization of adnexal masses in this study. Despite their high sensitivity (88.2%, 100%) they showed low Specificity (50%, 30%) respectively. Using the threshold criterion for WIR > 20.89, 5 benign cases showed false positive results. 2 tubo-ovarian abscesses (WIR 58.26 and 23.03 respectively) 2 fibrothecoma (WIR 56.14 and 67.15 respectively) and 1 sclerosing stromal tumor (WIR 33.85). This is inconsistent with Bernardin et al. who found a significant difference between the WIR of benign and malignant adnexal masses. Applying a cut-off WIR of 9.5 l/s, there was a sensitivity of 67% and specificity of 88% in predicting borderline/invasive malignancy (3). The disagreement could be attributed to the different pathological distribution of both studies. In this study, it has been shown that WIR is the most sensitive (88.2%) parameter while WOR is the most specific(. The combined ROC curve analysis of both WIR and WOR revealed improved sensitivity over WOR alone and greater specificity when compared to WIR alone. Thus, we can depend upon WIR in depicting malignant masses and correct our diagnosis by applying the cutoff of WOR so avoiding radical surgery in unnecessary patients (**Figure 5**). Applying a TPP cut off value of 143.28 gave high Specificity (80%) with high PPV 80% however it showed low sensitivity 47.1% hindering its diagnostic accuracy.

The current study had some limitations, for instance the limited number of ovarian epithelial borderline cases so we couldn’t estimate cut off value to discriminate between borderline and malignant cases. Ovarian borderline tumors occur mostly in patients of reproductive age and a conservative rather than radical surgical approach may be recommended to preserve fertility with an excellent prognosis. This restricted dataset may influence the reliability of the suggested cutoff values so further studies with larger sample numbers and better pathological distribution are recommended. Additionally, there are certain pitfalls in using the external myometrium as reference point that the myometrium must be at the same acquired slice as the enhancing component of the adnexal lesion, which is not always possible. In this situation oblique planes can be used. In addition, using MR imaging signal intensity is mostly affected by the type of acquisition parameters, such as flip angle and TR. Also, the linearity between signal intensity variation and contrast agent concentration is highly dependent on these parameters. Thus, many authors argue that we need to obtain reproducible perfusion parameters independent of acquisition conditions. Developing perfusion parameters expressed according to gadolinium concentration rather than signal intensity would address this issue. Likewise, a more recent approach consists in a quantitative analysis which is based on pharmacokinetic modeling allowing the conversion of signal intensity into gadolinium concentration. This necessitates a faster DCE acquisition and the use of a specialized workstation.

## Conclusion

The addition of DCE MR Sequence to the conventional MRI has improved its diagnostic accuracy. It provides additional information for tumor vascularity. In the current study, the most perfusion parameter that showed significant difference between benign and malignant lesions was WOR. We demonstrated that a lesion with a WOR > 6.03 is likely to be malignant with PPV 93.3%. WIR showed high sensitivity (88.2%) but low Specificity (50%). Thus, we can depend upon WIR in depicting malignant masses and correct our diagnosis by applying the cutoff of WOR to exclude the false positive cases and avoid unnecessary radical surgery especially in women aiming at preserving fertility. SI max showed relevant criterion for distinguishing invasive from non- invasive tumors as it represents the vascular density within the tumor. In our study it showed 73.2% accuracy and PPV 81.2% in distinguishing benign and malignant tumors.

## Data Availability

The raw data supporting the conclusions of this article will be made available by the authors, without undue reservation.

## References

[1] Foti PV Attinà G Spadola S Caltabiano R Farina R Palmucci S . MR imaging of ovarian masses: classification and differential diagnosis. Insights Imaging. (2016) 7:21–41. doi: 10.1007/s13244-015-0455-4. PMID: 26671276 PMC4729709

[2] Bazot M Nassar-Slaba J Thomassin-Naggara I Cortez A Uzan S Daraï E . MR imaging compared with intraoperative frozen-section examination for the diagnosis of adnexal tumors: correlation with final histology. Eur Radiol. (2006) 16:2687–99. doi: 10.1007/s00330-006-0163-z. PMID: 16547708

[3] Bernardin L Dilks P Liyanage S Miquel ME Sahdev A Rockall A . Effectiveness of semiquantitative multiphase dynamic contrast-enhanced MRI as a predictor of Malignancy in complex adnexal masses: radiological and pathological correlation. Eur Radiol. (2012) 22:880–90. doi: 10.1007/s00330-011-2331-z. PMID: 22095438

[4] Chu LC Coquia SF Hamper UM . Ultrasonography evaluation of pelvic masses. Radiol Clin North Am. (2014) 52:1237–52. doi: 10.1016/j.rcl.2014.07.003. PMID: 25444103

[5] Thomassin-Naggara I Bazot M Daraï E Callard P Thomassin J Cuenod CA . Epithelial ovarian tumors: value of dynamic contrast-enhanced MR imaging and correlation with tumor angiogenesis. Radiology. (2008) 248:148–59. doi: 10.1148/radiol.2481071120. PMID: 18458244

[6] Thomassin-Naggara I Balvay D Rockall A Carette MF Ballester M Daraï E . Added value of assessing adnexal masses with advanced MRI techniques. BioMed Res Int. (2015) 2015:1–10. doi: 10.1155/2015/785206. PMID: 26413542 PMC4564594

[7] Kazerooni AF Malek M Haghighatkhah H Parviz S Nabil M Torbati L . Semiquantitative dynamic contrast-enhanced MRI for accurate classification of complex adnexal masses. J Magn Reson Imaging. (2017) 45:418–27. doi: 10.1002/jmri.25359. PMID: 27367786

[8] Bazot M Haouy D Daraï E Cortez A Dechoux-Vodovar S Thomassin-Naggara I . Is MRI a useful tool to distinguish between serous and mucinous borderline ovarian tumours? Clin Radiol. (2013) 68:e1–9. doi: 10.1016/j.crad.2012.08.021. PMID: 23044365

[9] Thomassin-Naggara I Toussaint I Perrot N Rouzier R Cuenod CA Bazot M . Characterization of complex adnexal masses: value of adding perfusion- and diffusion-weighted MR imaging to conventional MR imaging. Radiology. (2011) 258:793–803. doi: 10.1148/radiol.10100751. PMID: 21193596

[10] Forstner R Thomassin-Naggara I Cunha TM Kinkel K Masselli G Kubik-Huch R . ESUR recommendations for MR imaging of the sonographically indeterminate adnexal mass: an update. Eur Radiol. (2017) 27:2248–57. doi: 10.1007/s00330-016-4600-3. PMID: 27770228 PMC5408043

[11] El Ameen NF Eissawy MG Mohsen LAMS Nada OM Beshreda GM . MR diffusion versus MR perfusion in patients with ovarian tumors: how far could we get? Egypt J Radiol Nucl Med. (2020) 51:1–11. doi: 10.1186/s43055-020-00170-5. PMID: 38164791

[12] Thomassin-Naggara I Daraï E Cuenod CA Rouzier R Callard P Bazot M . Dynamic contrast-enhanced magnetic resonance imaging: a useful tool for characterizing ovarian epithelial tumors. J Magn Reson Imaging. (2008) 28:111–20. doi: 10.1002/jmri.21377. PMID: 18581400

[13] Tomao F Di Pinto A Sassu CM Di Donato V Muzii L Petrella MC . Fertility preservation in ovarian tumors. Ecancermedicalscience. (2018) 12:885. doi: 10.3332/ecancer.2018.885. PMID: 30679952 PMC6345054

[14] Thompson EM Guillaume DJ . Perfusion MRI: technical aspects. AJR Am J Roentgenol. (2013) 200:24–34. 23255738 10.2214/AJR.12.9543 PMC3593114

[15] Gundogdu S Erdem CZ Erdem LO Bayar U . Enhancement kinetics of normal ovaries on dynamic contrast-enhanced MR imaging. Eur J Obstet Gynecol Reprod Biol. (2006) 129:60–4. doi: 10.1016/j.ejogrb.2006.03.017. PMID: 16698167

[16] Thomassin-Naggara I Balvay D Cuenod CA Daraï E Marsault C Bazot M . Dynamic contrast-enhanced MR imaging to assess physiologic variations of myometrial perfusion. Eur Radiol. (2010) 20:984–94. doi: 10.1007/s00330-009-1621-1. PMID: 19820949

[17] Dilks P Narayanan P Reznek R Sahdev A Rockall A . Can quantitative dynamic contrast-enhanced MRI independently characterize an ovarian mass? Eur Radiol. (2010) 20:2176–83. doi: 10.1007/s00330-010-1795-6. PMID: 20419493

[18] Jin J Deng X Long L Liu M Cao M Gong H . The value of a radiomics model in predicting ovarian Malignancy: a retrospective multi-center comparison with O-RADS and radiologists. Insights Imaging. (2025) 16:163–76. doi: 10.1186/s13244-025-02047-w. PMID: 40745233 PMC12314133

[19] Elkady RM . Radiomics analysis in evaluation of cervical cancer: a further step on the road. Acad Radiol. (2022) 29:1141–42. doi: 10.1016/j.acra.2022.02.018. PMID: 35307261

[20] Lugano R Ramachandran M Dimberg A . Tumor angiogenesis: causes, consequences, challenges and opportunities. Cell Mol Life Sci. (2020) 77:1745–70. doi: 10.1007/s00018-019-03351-7. PMID: 31690961 PMC7190605

[21] Barrett T Brechbiel M Bernardo M Choyke PL . MRI of tumor angiogenesis. J Magn Reson Imaging. (2007) 26:235–49. doi: 10.1002/jmri.20991. PMID: 17623889

[22] Liberman L Morris EA Lee MJY Kaplan JB LaTrenta LR Menell JH . Breast lesions detected on MR imaging: features and positive predictive value. AJR Am J Roentgenol. (2002) 179:171–78. doi: 10.2214/ajr.179.1.1790171. PMID: 12076929

[23] Kuhl CK Mielcareck P Klaschik S Leutner C Wardelmann E Gieseke J . Dynamic breast MR imaging: are signal intensity time course data useful for differential diagnosis of enhancing lesions? Radiology. (1999) 211:101–10. doi: 10.1148/radiology.211.1.r99ap38101. PMID: 10189459

[24] Jabbar SB Lynch B Seiler S Hwang H Sahoo S . Pathologic findings of breast lesions detected on magnetic resonance imaging. Arch Pathol Lab Med. (2017) 141:1513–22. doi: 10.5858/arpa.2016-0552-OA. PMID: 28782985

[25] El Backry M Shady M Mousa AE Zaky MM . Role of dynamic contrast-enhanced MR perfusion in differentiation between benign and Malignant tumors. Egypt J Radiol Nucl Med. (2015) 46:715–26. doi: 10.1016/j.ejrnm.2015.01.001. PMID: 38826717

[26] Bizeul J Ronot M Roux M Cannella R Lebigot J Aubé C . Evaluation of washout using subtraction MRI for the diagnosis of hepatocellular carcinoma in cirrhotic patients with spontaneously T1-hyperintense nodules. Diagn Interv Imaging. (2023) 104:427–34. doi: 10.1016/j.diii.2023.04.005. PMID: 37120391

[27] Jansen SA Fan X Karczmar GS Abe H Schmidt RA Newstead GM . Differentiation between benign and Malignant breast lesions detected by bilateral dynamic contrast-enhanced MRI: a sensitivity and specificity study. Magn Reson Med. (2008) 59:747–54. doi: 10.1002/mrm.21530. PMID: 18383287 PMC3121098

[28] Abe H Mori N Tsuchiya K Schacht DV Pineda FD Jiang Y . Kinetic analysis of benign and Malignant breast lesions with ultrafast dynamic contrast-enhanced MRI: comparison with standard kinetic assessment. AJR Am J Roentgenol. (2016) 207:1159–66. doi: 10.2214/AJR.15.15957. PMID: 27532897 PMC6535046

[29] Schnall MD Rosten S Englander S Orel SG Nunes LW . A combined architectural and kinetic interpretation model for breast MR images. Acad Radiol. (2001) 8:591–97. doi: 10.1016/S1076-6332(03)80683-9. PMID: 11450959

[30] Sohaib SA Sahdev A Van Trappen P Jacobs IJ Reznek RH . Characterization of adnexal mass lesions on MR imaging. AJR Am J Roentgenol. (2003) 180:1297–304. doi: 10.2214/ajr.180.5.1801297. PMID: 12704041

[31] Gity M Parviz S Rad HS Kazerooni AF Shirali E Shakiba M . Differentiation of benign from Malignant adnexal masses by dynamic contrast-enhanced MRI (DCE-MRI): quantitative and semi-quantitative analysis at 3-Tesla MRI. Asian Pac J Cancer Prev. (2019) 20:1073–79. doi: 10.31557/APJCP.2019.20.4.1073. PMID: 31030476 PMC6948906

