# Supplementary material for: MRI perfusion in evaluating ovarian masses: diagnostic performance of wash out rate
Source: Front Oncol. 2026 May 28;16:1609090. doi: 10.3389/fonc.2026.1609090 (PMC13253287; doi:10.3389/fonc.2026.1609090)
Supplement: Supplementary file 1 [file Table1.docx]

S1: Semiquantitative analysis parameters; S0 is the initial intensity, S1 is the peak intensity, T0 is the Time of Arrival (time of initial intensity), T1 is the time of peak intensity.


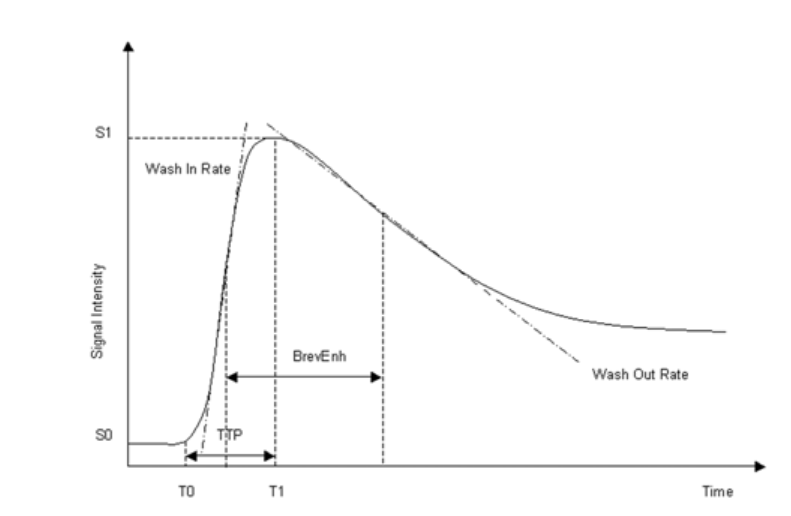


**Relative Enhancement [%] (RELENH)**

The signal enhancement of a pixel of certain dynamic relative to that same pixel in the reference dynamic. The reference dynamic is normally the first, pre-contrast dynamic.

**Maximum Enhancement (MAXENH)**

Difference between peak intensity S1 and S0.

**Maximum Relative Enhancement [%] (MAXRELENH)**

Maximum of all relative enhancements over all dynamics

(SIrel)=(SImax−SI0)/ SI0×100

**T0 - Time of Arrival [s] (T0)**

Arrival of the contrast agent, i.e. Begin of the enhancement curve.

**Time to Peak (TTP)**

Time between T0 and the time of peak intensity (T1).

**Wash in Rate [l/s] (WASHIN}**

Maximum slope between T0 and time of peak intensity T1.

SI0−SImax/time(s)

**Wash out Rate [l/s] (WASHOUT)**

Maximum slope between time of peak intensity T1 and the end of the

Measurement.

**Brevity of Enhancement [s] (BREVENH)**

Time between point of maximum wash in rate and maximum wash out Rate.

**Area under the curve (AREACURV)**

Sum of all intensities under the curve.
